# Targeting DNA Replication Stress for Cancer Therapy

**DOI:** 10.3390/genes7080051

**Published:** 2016-08-19

**Authors:** Jun Zhang, Qun Dai, Dongkyoo Park, Xingming Deng

**Affiliations:** 1 Division of Hematology, Oncology and Blood & Marrow Transplantation, Department of Internal Medicine, Holden Comprehensive Cancer Center, University of Iowa Carver College of Medicine, 200 Hawkins Drive, Iowa City, IA 52242, USA; qun-dai@uiowa.edu; 2 Division of Cancer Biology, Department of Radiation Oncology, Emory University School of Medicine and Winship Cancer Institute of Emory University, 1365C Clifton Road NE, Atlanta, GA 30322, USA; dpark33@emory.edu

**Keywords:** DNA replication stress, cancer, targeted therapy

## Abstract

The human cellular genome is under constant stress from extrinsic and intrinsic factors, which can lead to DNA damage and defective replication. In normal cells, DNA damage response (DDR) mediated by various checkpoints will either activate the DNA repair system or induce cellular apoptosis/senescence, therefore maintaining overall genomic integrity. Cancer cells, however, due to constitutive growth signaling and defective DDR, may exhibit “replication stress” —a phenomenon unique to cancer cells that is described as the perturbation of error-free DNA replication and slow-down of DNA synthesis. Although replication stress has been proven to induce genomic instability and tumorigenesis, recent studies have counterintuitively shown that enhancing replicative stress through further loosening of the remaining checkpoints in cancer cells to induce their catastrophic failure of proliferation may provide an alternative therapeutic approach. In this review, we discuss the rationale to enhance replicative stress in cancer cells, past approaches using traditional radiation and chemotherapy, and emerging approaches targeting the signaling cascades induced by DNA damage. We also summarize current clinical trials exploring these strategies and propose future research directions including the use of combination therapies, and the identification of potential new targets and biomarkers to track and predict treatment responses to targeting DNA replication stress.

## 1. Introduction

Accurate DNA replication in dividing cells is crucial to maintaining the integrity of the human genome. To ensure the accuracy of DNA replication, DNA damage response (DDR) mediated by various cell cycle checkpoints either activates the DNA repair system or induces cellular apoptosis/senescence when DNA damage arises—which is almost inevitable considering the array of stresses from intrinsic and extrinsic factors. When there is a loss of or defect in DDR due to oncogenic activation or tumor suppressor inactivation, DNA replication may persist to meet the demands of unrestrained proliferation despite the presence of unrepaired DNA lesions, which then leads to “replication stress”—a phenomenon unique to cancer cells that is described as the perturbation of error-free DNA replication and slow-down of DNA synthesis. Replication stress induces genomic instability and therefore potentiates oncogenic transformation. However, the novel concept of further enhancing replication stress may provide a plausible alternative to treat cancer due to the induction of “mitotic catastrophe”.

Here, we review the mechanisms underlying replication stress and the endeavors of researchers to harness this phenomenon for cancer treatment, with specific focus on emerging approaches with promising preclinical/clinical data. We also propose novel ideas including the identification of future targets as well as biomarkers to track and predict treatment response.

### 1.1. Replication Stress and Its Underlying Mechanisms

Extrinsic insults such as irradiation and genotoxic agents, or intrinsic stress such as reactive oxygen species (ROS) and misincorporation of nucleotides can all induce DNA damage [1,2]. In normal cells, these DNA errors are fixed by repair mechanisms, and if not, cell proliferation is halted and cell death often ensues [1]. With defective DDR and/or loss of cell cycle checkpoints, which occur along with sustained growth signaling, cells may still manage to replicate damaged DNA to meet the demands of unrestrained proliferation [3]. When doing so, the DNA polymerases at the replication forks temporarily cease their activity resulting in a phenomenon called “fork stalling” [4]. These stalled “forks” activate the replicative mini-chromosome maintenance (MCM) to continue unwinding DNA for a few hundred base pairs downstream, thereby exposing single-stranded DNA (ssDNA) [1,4]. ssDNAs then activate the ATR signaling cascade, manifest as the phosphorylation of checkpoint kinase 1 (Chk1), cell cycle checkpoint RAD17 and histone H2AX. These events are collectively described as “replicative stress” [1,3,4].

Replicative stress seems to be unique to cancer cells since it is rarely observed in normal cells even when they proliferate rapidly [3]. It is postulated that oncogene activation can stimulate the G1-S cell cycle transition, resulting in premature onset of S phase and therefore insufficient levels of DNA replicative enzymes and/or nucleotides, which are prerequisites for accurate DNA replication [5,6]. Conversely, the inactivation of key tumor suppressors, such as TP53, RB1 and CDKN2A etc., may also induce replicative stress by promoting G1-S transition [7]. In addition, cancer cells typically have higher levels of ROS due to increased MYC activity [8], enhanced production by mitochondria [9], or hypoxia owing to relatively insufficient vascularization [10]. The accumulation of ROS can lead to the formation of 8-oxoguanine that causes base pair mismatch [11]. Finally, tumor cells often lack efficient DNA repair systems, for example, secondary to the loss of BRCA1 [12,13]. All these factors contribute to the development of replication stress—a unique feature in cancer cells that can theoretically serve as a therapeutic target.

### 1.2. Rationale for Enhancing Replication Stress to Cause Cancer Cell Death

**Figure 1:**
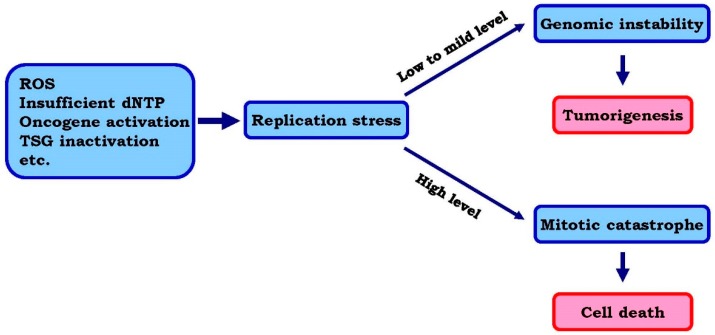
Rationale for enhancing replication stress to kill cancer cells. DNA replicative stress can be induced by various factors including ROS, insufficient dNTP, oncogene activation or the loss/inactivation of tumor suppressors, etc. At the low to mild level, the replicative stress predominantly induces genomic instability, therefore facilitates tumorigenesis and cancer progression. However, when the replicative stress is enhanced to a high level through further loss of checkpoints, cancer cells may enter the mitotic phase with incomplete or uncorrected DNA replication, which eventually leads to cell death through mitotic catastrophe. Therefore, enhancing replicative stress can be a novel approach to kill cancer cells. TSG: Tumor suppressing gene.

The net effect of replicative stress is the stalling of replication forks and the accumulation of ssDNA as previously described [1,3,4]. The ssDNA is rapidly coated by ssDNA-binding proteins such as replication protein A (RPA), which then activates the ATR signaling pathway resulting in the subsequent phosphorylation of Chk1 kinase [1,14]. The activity of this pathway is crucial to stabilize stalled forks as well as activate cell cycle S-M checkpoints so as to limit entry into mitosis in the presence of unreplicated DNA [15]. However, under conditions that enhance replication stress, such as in the absence of ATR and Chk1, stalled forks can persist and other replication origins are fired, leading to an exhaustion of deoxynucleotide triphosphate (dNTP) pools, which aggravate replicative stress and further accumulation of non-progressive forks [16]. When the amount of ssDNA exceeds the amount of available RPA, the forks may collapse leading to the generation of DNA double strand breaks (DSBs) [4,16]. When these cells are allowed to enter mitosis, the unreplicated chromosomes will trigger cell death through mitotic catastrophe [16,17]. In fact, although replication stress induces genomic instability and fuels tumorigenesis, studies have shown this is only the case when replicative stress occurs at low to mild levels [1,3,18]. High levels of replicative stress, however, may instead induce cancer cell death through mitotic catastrophe and therefore counteract cancer progression [1,3,18] (Figure 1).

To enhance replication stress, several key steps are theoretically subject to manipulation: the entry into S phase; DNA synthesis, replication and repair; and the premature entry into M phase. We will consider conventional approaches such as chemotherapy and radiation, and extend our discussion to emerging novel strategies to enhance replication stress.

## 2. Approaches to Harness Replication Stress for the Treatment of Cancer

### 2.1. Conventional Approaches

#### Radiation and chemotherapy

**Figure 2:**
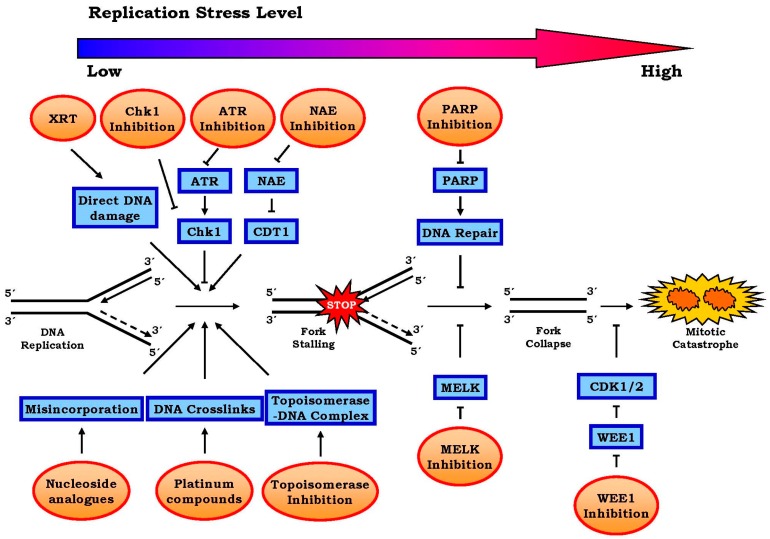
Illustration of various approaches to target replication stress for cancer treatment. For simplicity, only the approaches that have been discussed in the text are illustrated here. As indicated, with the progression from DNA replication fork stalling to folk collapse and eventually premature entry of mitotic phase, there is accompanying enhancement of DNA replication stress. Shown here are different approaches exploited to enhance this stress level. While chemotherapeutic agents use different approaches to induce fork stalling (e.g., nucleotide disincorporation, DNA crosslinks and topoisomerase—DNA complex), radiation (XRT) induces direct DNA damage. These genetic errors activate ATR-Chk1 signaling which prevents further fork stalling. Therefore, inhibitors of either ATR or Chk1 may enhance replicative stress. Since both PARP and MELK prevent the progression to fork collapse, their corresponding inhibitors may also augment the level of replicative stress. Because the ubiquitin ligase substrate CDT1 causes DNA to replicate more than once and its activity is inhibited by neddylation, the NAE inhibitor can also be used to achieve this purpose. Finally, WEE1 inhibitor activates CDK1/2, therefore facilitating premature entry to S phase. The final consequence of all these approaches is cell death through mitotic catastrophe that is induced by the enhancement of replicative stress.

While radiation induces DNA damage directly by creating ssDNA and DSBs that directly interfere with DNA replication [19], chemotherapy enhances replicative stress in various ways. For example, alkylating agents (e.g., cyclophosphamide and temozolomide) and platinum compounds (e.g., cisplatin and carboplatin) can modify DNA to produce intra- and inter-strand crosslinks between nucleotide bases [20,21]. Intra-strand crosslinks induce DNA lesions in the template strand as well as misincorporate nucleotides [22], and inter-strand crosslinks induce defects in DNA unwinding—the very first step of DNA replication [23]. Apparently, these crosslinks will delay the progression of replication forks and enhance replicative stress (Figure 2).

Nucleoside and base analogues act in a different way. They tend to reduce the pool of dNTPs—the building block of DNA strands—and therefore result in a shortage of replication materials/factors. As an example, the chemotherapeutic agent gemcitabine inhibits ribonucleotide reductase, whereas 5-fluorouracil (5-FU) inhibits thymidylate synthetase. Both agents reduce the size of the available dNTP pools that are needed for DNA synthesis and therefore induce stalling of replication forks [24,25] (Figure 2). Topoisomerase inhibitors also enhance replicative stress. The topoisomerases control DNA supercoiling and entanglement by catalyzing nicking and re-ligation of DNA strands [26]. By forming complexes with topoisomerases when they bind to DNA, topoisomerase inhibitors can physically hinder ongoing replication forks [27,28]. In addition, topoisomerase I inhibitors (e.g., irinotecan and topotecan) may also induce fork reversal [29], and topoisomerase II inhibitors (e.g., etoposide and doxorubicin) may inactivate Chk1 [30], an important checkpoint that we will address in detail below.

### 2.2. Emerging Approaches

Distinct from the traditional radiation and chemotherapy that induce replicative stress more or less through affecting the integrity of DNA directly, several emerging approaches target signaling cascades that are induced by DNA damage. The following represent promising novel interventions to enhance replicative stress.

#### 2.2.1. Targeting ATR-Chk1 Signaling

As mentioned, ssDNA triggers ATR-Chk1 signaling, which has a crucial role in suppressing replicative stress [1,3,4]. It appears excessive replication stress is deleterious even in cancer cells as they cannot complete mitosis with unreplicated regions of genome, and will undergo mitotic catastrophe if replication stress persists into mitosis [16]. Cancer cells therefore need to maintain a proficient response system, such as the ATR-Chk1 pathway, to cope with the high level of replicative stress [16,17]. In addition, Chk1 suppresses CDK activity to secure the orderly activation of replication origins during S phase [31]. It is therefore logical to use inhibitors targeting ATR-Chk1 signaling to further enhance replicative stress. Studies have shown that with the inhibition or absence of Chk1, DNA replication is inappropriately initiated from multiple origins, leading to the exhaustion of replication factors and to fork stalling and collapse [32,33]. Currently, several Chk1 inhibitors are being evaluated in phase I and II clinical trials [34,35,36] (Table 1).

**Figure 3:**
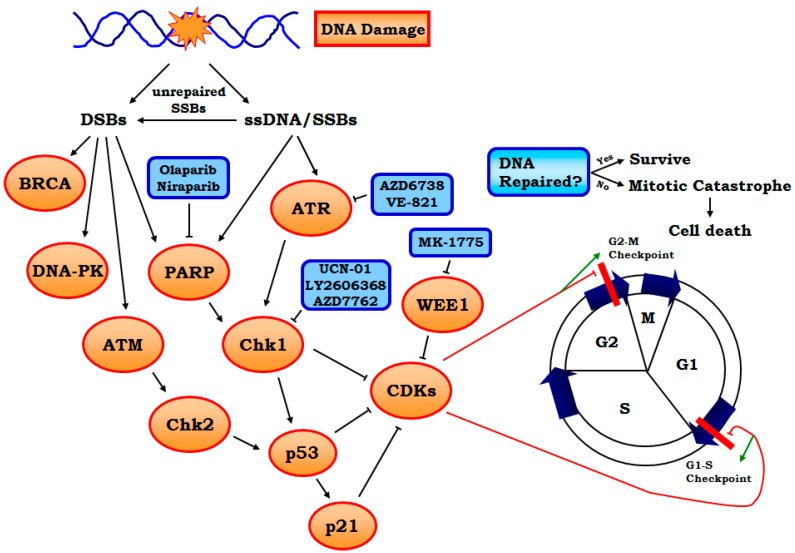
Current and potential targeting strategies in association with the cell cycle. Shown here are two major checkpoints, G1/S and G2/M. The G2/M checkpoint is crucial to induce cell cycle arrest and allow the cell to repair DNA defects before it enters M phase. Both checkpoints, however, can be inhibited by CDKs which facilitate cell cycle progression regardless of the DNA defects. Theoretically, if the cell is allowed to enter mitosis without DNA damage properly fixed, cell death could happen due to mitotic catastrophe. Therefore, in the setting of replicative stress, approaches facilitating cell cycle progression through G2-M, such as activating CDKs or reducing the inhibitory effect on CDKs could have a cell killing effect. DNA damage will result in both DSBs and ssDNA/SSBs, with the latter as the major cause of replicative stress. ssDNA/SSBs activate ATR-Chk1 signaling, which in turn activates tumor suppressors p53 and p21 and inhibits CDKs. ssDNA/SSBs may also activate PARP which in turn enhances Chk1 activity. Wee1 can directly inhibit CDKs. Therefore, inhibitors of PARP, ATR, Chk1 and Wee1 will all activate CDKs and allow the cell to enter mitosis despite the presence of unrepaired DNA. If SSBs are not fixed, they may become secondary DSBs, therefore targeting DSB-induced DNA repair mechanisms could also be a promising strategy. DSBs activate the tumor suppressor BRCA as well as ATM-Chk2 signaling and DNA-PK. Chk2 also activates p53. Therefore, it is not surprising that inhibition of ATR-Chk1 could be particularly useful for p53 deficient tumors, and PARP inhibition for patients with BRCA mutation. (DSBs: Double strand breaks; SSBs: Single strand breaks; DNA-PK: DNA-dependent protein kinase; CDK: Cyclin-dependent kinase).

Since Chk1 activity is strongly enhanced by ATR-mediated phosphorylation, the inhibition of ATR should theoretically produce a similar effect [37,38]. In fact, specific ATR inhibitors, such as AZD6738 and VE-821, have been shown to be effective in the preclinical setting, and are now in clinical trials for both solid tumor and hematological malignancies [37,39] (Table 1). Interestingly, reduced ATR activity has been shown to have a synthetic lethality effect in combination with the loss of p53, suggesting that the inhibition of ATR could be particularly useful for the treatment of p53-deficient tumors [38]. This is not surprising since ATR can activate p53 (Figure 3).

#### 2.2.2. PARP1 Inhibitors

Poly (ADP-ribose) polymerase 1 and 2 (PARP) are nuclear proteins that are activated by DSBs [51]. The function of PARP1 has been well studied; it protects DNA breaks and chromatin structure and recruits DNA repair and checkpoint proteins to the sites of damage [52,53]. A recent study has demonstrated that PARP1 binds to and is activated at stalled replication forks to mediate recruitment of Mre11 to initiate the end processing required for replication restart [53]. PARP1 also enhances the activation of Chk1 [54] (Figure 3). Therefore, inhibition of PARP1 enhances replicative stress. In addition, inhibition of PARP1 was found to be synthetically lethal for cells with defects in homologous recombination (HR) [55], a DSBs repair mechanism, and was particularly effective in tumor cells that lack functional BRCA1 or BRCA2 [55,56,57,58]. The function of PARP2 is largely unknown. However, it shares homology with PARP1 and it may compensate for the function of PARP1 based on observations using animal models of embryonic knockout of PARP1 and/or PARP2 [59,60].

Recent studies have suggested several reasons for the synthetic lethality observed in the setting of BRCA or HR deficiency when PARP1 inhibition is applied: (1) The persistent accumulation of single strand DNA breaks (SSBs) leads to their eventual conversion to DSBs, therefore, the mechanism of intact DSBs repair is crucial. DSBs repair depends on HR and non-homologous end joining (NHEJ). Since PARP1 is involved in NHEJ [61], cancer cells with deficiency in BRCA or HR will thus require PARP1-dependent NHEJ for DSBs repair, and become more vulnerable to apoptosis when PARP1 is inhibited [55]; (2) Both BRCA2 and PARP1 are important in protecting stalled replication forks from degradation [62]. BRCA2 deficient cells are therefore more sensitive to PARP1 inhibition; (3) Chemical PARP1 inhibitors cause the PARP1 enzyme to be trapped on the DNA, therefore hindering the access of DNA repair proteins [63]. If the DNA repair function of HR is intact, the inhibition of PARP1 alone may not necessarily induce cell death, however, killing is facilitated when there is a deficiency in BRCA or HR; (4) Due to replicative stress, increased PARP1 activity is required. Therefore, HR becomes essential to repair DNA damage upon PARP1 inhibition. When there is a deficiency in BRCA or HR, synthetic lethality thus ensues [55].

PARP1 inhibitors such as olaparib and niraparib have been tested in clinical trials in breast and ovarian cancers [44,45,58]. Recently, based on an open-label, non-randomized clinical trial that enrolled 137 patients with measurable, germline BRCA mutation (gBRCAm)-associated ovarian cancer [43], olaparib was approved by the FDA as a monotherapy for the treatment of patients with deleterious or suspected deleterious gBRCAm advanced ovarian cancer who have been treated with three or more prior lines of chemotherapy [43]. PARP inhibition is also promising for triple negative breast cancer (TNBC), which is an aggressive breast cancer entity that lacks targeted therapy [64]. TNBC is believed to have high levels of replicative stress due to c-MYC amplification and EGFR activation [65]. More importantly, reports to date indicate that up to 20% of TNBC patients harbor germline BRCA mutations [66]. Even in patients with sporadic TNBC without BRCA mutation, a proportion of them share BRCA1 mutation-like tumor characteristics (aka “BRCAness”) where BRCA is inactivated by other means such as promoter methylation [67]. Because of impaired HR from either BRCA mutation or BRCAness, PARP inhibition is believed to be a rational approach for this subtype of breast cancer [64]. In fact, in phase II studies, olaparib did not result in objective responses in non-BRCA-associated TNBC [68], however, a significant effect was observed in breast cancer patients with BRCA mutation [69]. In addition, using a patient derived xenograft (PDX) model, inhibition of EGFR using ^177^ Lu-labelled anti-EGFR monoclonal antibody in combination with chemotherapy and PARP inhibition successfully eradicated putative breast cancer stem cells [70]. A similar synergistic effect was observed in another study when the EGFR/HER2 tyrosine kinase inhibitor lapatinib was co-administered with PARP inhibitor veliparib to induce persistent DSBs in TNBC [71]. Lapatinib was found not only to induce HR deficiency but also to sequester BRCA1 away from its nuclear repair substrates [71]. These observations suggest a potential synthetic lethality when inhibition of EGFR and PARP are combined in TNBC.

Other than using BRCA mutation and BRCAness status, recent advancements in large-scale genomics and sequencing studies will likely help in identifying additional candidates for PARP inhibition. For example, Alexandrov et al. was able to re-classify tumors based on mutation signatures, among which one particular signature in breast, ovarian and pancreatic cancer was associated with inactivating mutations of either BRCA1 or BRCA2 [72]. Interestingly, some cases with this particular signature did not have BRCA mutations, suggesting other BRCA inactivating mechanisms exist or other genetic abnormalities could confer a similar phenotype [72]. These cases could also potentially benefit from PARP inhibition. Similarly, using whole-genome sequencing to extend the signature analysis to genome rearrangements, Nik-Zainal et al. recently reported three rearrangement signatures that are associated with defective HR, including two that involve either BRCA1 or BRCA2 deficiency [73], therefore providing another approach to identify patients that could potentially benefit from PARP inhibition or other therapies targeting deficient HR mechanism.

#### 2.2.3. Other Targets Including WEE1, MELK and Neddylation, etc.

WEE1 is a nuclear serine/threonine kinase that inhibits cell entry into mitosis through inhibiting CDK1 and CDK2 [74]. When WEE1 is inhibited by drugs, CDK activity is enhanced and cells in S phase can be induced to enter mitosis prematurely even if DNA replication is defective or incomplete [74,75] (Figure 3). The increased CDK activity after WEE1 inhibition also rapidly increases replication initiation, leading to a shortage of nucleotides that are required for DNA replication [75]. WEE1 inhibitors can thus be powerful tools to enhance replicative stress and drive cells undergoing a high level of this stress into premature mitosis and subsequent death from mitotic catastrophe [46] (Table 1).

The maternal embryonic leucine zipper kinase (MELK) is another serine/threonine kinase that plays an important role in cell cycle control [76]. Recently, in glioma cells, inhibition or knockdown of MELK was found to increase the accumulation of stalled replication forks and induce a DNA damage response during S phase [77]. Therefore, MELK inhibitors hold promise as enhancers of replicative stress in cancer cells, and some of them are now in clinical phase I trials [48,49] (Table 1).

Replicative stress can also be reduced by neddylation, a ubiquitin-like modification [78]. Neddylation is a prerequisite for the activity of cullin-like ubiquitin ligases [79]. Therefore, when neddylation is inhibited, the substrates of these ubiquitin ligases accumulate. Among the substrates, the DNA replication factor CDT1 forms a complex with the replication-licensing factor geminin, causing DNA to replicate more than once per S phase, therefore rapidly consuming dNTP [80,81]. Neddylation can be inhibited by targeting NEDD8-activating enzyme (NAE), a ubiquitin-like protein, using MLN4924 [82,83,84], which has just completed a phase I clinical trial [50] (Table 1).

## 3. Future Directions

### 3.1. Potential of Combination Therapy

Since different replication-enhancing agents work through different mechanisms, it is worth exploring the potential of combination approaches. In fact, the combination of conventional treatments has been utilized for decades, for example, the combination of chemotherapy and radiation in the neoadjuvant or adjuvant setting, or as a definitive therapeutic strategy for certain types of cancer [85,86]. Combinations of different chemotherapeutic agents have also been well studied and some have become components of classical chemotherapy regimens for certain cancers, for example, the combination of platinum compound cisplatin or carboplatin with topoisomerase inhibitor etoposide for small cell lung cancer [87,88]. However, more studies are needed to explore the optimal combinations of conventional and emerging approaches, as well as the combination of different novel strategies.

Studies have shown enhanced efficacy of gemcitabine when combined with inhibitors of Chk1, ATR, WEE1, or NAE [47,89,90]. However, a Phase I clinical trial combining Chk1 inhibitor AZD7762 with gemcitabine revealed unexpected cardiotoxicity [41,42], necessitating the search for alternative combinations. There is also preclinical data supporting a potential synergistic effect when platinum compounds are used in combination with inhibitors of Chk1, ATR, and WEE1, etc. [74,91,92], but this needs to be explored further in clinical trials. Targeted inhibitors may also be combined with each other. For example, the combination of Chk1 and WEE1 inhibitors was found to enhance replicative stress and promote mitotic catastrophe [93,94,95]. Future studies are needed to identify promising combinations among these targeted inhibitors, and to determine their underlying mechanisms and clinical usage.

### 3.2. Identification of Future Targets

One approach that could be exploited in the future is to increase ROS levels because the incorporation of oxidized nucleotides enhances replicative stress [96]. Since oxidative stress is already augmented in tumors [97], its further enhancement could theoretically offer a selective target advantage over normal cells. It has been speculated that increasing ROS in cancer stem cells might make them more vulnerable to radiation therapy [98].

Besides ATR-Chk1 signaling, the ATM-Chk2 pathway and DNA-dependent protein kinase (DNA-PK) are also important targets activated by DNA replication stress, presumably due to secondary DSBs (Figure 3). Inhibitors of ATM-Chk2, DNA-PK and their downstream players could therefore potentiate replication stress [99,100]. Targeting other DNA repair systems, such as other players in homologous recombination repair (e.g., RAD51), seems to be a promising approach as well [101,102].

Other potential and provocative approaches that are worth mentioning include: (1) chromatin modification, e.g., using HDAC inhibitors to increase replicative stress [103]; (2) targeting various cell death pathways including apoptosis, senescence and autophagy since cell death is triggered by extensive replicative stress [104,105,106]; (3) promoting G1-S transition since replication stress is only possible during DNA synthesis (S phase) [107]. This approach is only theoretical at present and requires precise regulation of the timing to avoid persistent stimulation of cell proliferation.

### 3.3. Identification of Biomarkers to Detect and Track Replication Stress and Predict Treatment Response

It is important to identify biomarkers that allow accurate detection and reliable reflection of the degree of replicative stress. Currently available readouts such as H2AX immunohistochemical staining (IHC) do not distinguish between general DNA damage and specific replicative stress, and apoptosis measured by cleaved caspase or TUNEL reflect only the downstream consequences of replicative stress. Novel approaches are thus needed, for example, to detect the accumulation of ssDNA as determined by staining for bromodeoxyuridine (BrdU), or replication intermediates such as replication forks and incorporated nucleoside analogues [32,108]. It will be even more useful if we can detect such changes using circulating tumor cells in the peripheral blood, which hopefully can provide a convenient approach with which to monitor replicative stress during cancer therapy.

Since emerging targeted agents interfere with specific signaling pathways to enhance replicative stress, it is equally important to identify parameters that can predict therapeutic response, much like those for targeted therapies specific for receptor tyrosine kinases. For example, germline BRCA1/2 mutation status is crucial for the efficacy of PARP inhibitors, which are currently used only in a highly selected group of patients [43,45]. Since the strategy is to further increase replicative stress in a catastrophic manner, the p53 status and proliferation index, as assessed by Ki67, might serve as important markers, a theory that requires validation [38]. In addition, assessing chromosomal instability (CIN) by karyotype or measuring CIN genes might be another approach to predict response since CIN often results from replicative stress [109].

### 3.4. Identification of Resistance Mechanisms

Due to the fact that none of the therapies will work across all subtypes/subclasses of tumors, identification of the mechanisms of either intrinsic or acquired resistance is crucial to deliver better treatment. Using PARP inhibitor as an example, Lord et al. [110] provide a good summary of the resistant mechanisms including the acquisition of secondary BRCA mutations [111]; restoration of HR DNA repair activity through the loss of NHEJ factor 53BP1 [112,113]; upregulation of ATP-binding cassette (ABC) transporters such as p-glycoprotein efflux pump (PgP) [114]; and reduced PARP expression level [115,116], etc. Based on the potential role of ABC transporter in resistance, studies have demonstrated that coadministration of the PgP inhibitor tariquidar [114] or verapamil [117] could reverse the resistance to olaparib—underscoring the importance of investigating the resistance mechanisms. Similar studies have been carried out for ATR, Ch1 and Wee1 inhibitors [118,119].

## 4. Conclusions

In this article, we have discussed the mechanisms underlying DNA replication stress and approaches to exploit this process for cancer therapy. We have reviewed traditional strategies as well as emerging approaches/concepts to enhance replicative stress. Although some of these strategies are still in the experimental stage, enough evidence has accumulated to suggest that targeting DNA replication stress is not only promising but also selective for cancer cells. With greater understanding of DNA replication, we anticipate that the emergence of novel cancer therapies in this field will have great impact on cancer patients.

## Figures and Tables

**Table 1:** Therapeutic agents that enhance replicative stress.

Target/mechanism	Compounds	Stage of development	Reference
DNA misincorporation/modification	Alkylating agents e.g., cyclophosphamide, temozolamide, etc.	Approved	[20]
	Platinum compounds e.g., cisplatin, carboplatin, etc.	Approved	[21]
Ribonucleotide reductase	Gemcitabine	Approved	[24]
Thymidylate synthetase	5-fluorouracil	Approved	[25]
Topoisomerase I	Irinotecan, topotecan	Approved	[29]
Topoisomerase II	Etoposide, doxrubicin	Approved	[30]
Chk1	UCN-01	Phase I/II. Multiple studies completed	[34,40]
	LY2606368	Phase I/II, one study completed	[36]
	AZD7762	Phase I. One completed.	[41,42]
ATR	AZD6738	Phase I, one study completed	[37]
PARP1	Olaparib and niraparib	Approved	[43,44,45]
WEE1	MK-1775	Phase I	[46,47]
MELK	OTS167	Phase I, 2 studies	[48,49]
NAE	MLN4924	Phase I, multiple studies	[50]
